# Osteoporotic Bone Recovery by a Highly Bone‐Inductive Calcium Phosphate Polymer‐Induced Liquid‐Precursor

**DOI:** 10.1002/advs.201900683

**Published:** 2019-08-20

**Authors:** Shasha Yao, Xianfeng Lin, Yifei Xu, Yangwu Chen, Pengcheng Qiu, Changyu Shao, Biao Jin, Zhao Mu, Nico A. J. M. Sommerdijk, Ruikang Tang

**Affiliations:** ^1^ Center for Biomaterials and Biopathways Department of Chemistry Zhejiang University Hangzhou Zhejiang 310027 China; ^2^ Department of Orthopaedic Surgery Sir Run Run Shaw Hospital School of Medicine Zhejiang University Hangzhou Zhejiang 310016 China; ^3^ Key Laboratory of Musculoskeletal System Degeneration and Regeneration Translational Research of Zhejiang Province Hangzhou Zhejiang 310016 China; ^4^ Laboratory of Materials and Interface Chemistry and Center for Multiscale Electron Microscopy Department of Chemical Engineering and Chemistry Eindhoven University of Technology, Eindhoven PO box 513 5600 MB Eindhoven The Netherlands; ^5^ Institute for Complex Molecular Systems Eindhoven University of Technology, Eindhoven PO box 513 5600 MB Eindhoven The Netherlands; ^6^ Department of Orthopedic Surgery Second Affiliated Hospital School of Medicine Zhejiang University Hangzhou Zhejiang 310012 China

**Keywords:** bone recovery, bone‐inductive, mineralization, osteoporosis, polymer‐induced liquid‐precursor (PILP)

## Abstract

Osteoporosis is an incurable chronic disease characterized by a lack of mineral mass in the bones. Here, the full recovery of osteoporotic bone is achieved by using a calcium phosphate polymer‐induced liquid‐precursor (CaP‐PILP). This free‐flowing CaP‐PILP material displays excellent bone inductivity and is able to readily penetrate into collagen fibrils and form intrafibrillar hydroxyapatite crystals oriented along the *c*‐axis. This ability is attributed to the microstructure of the material, which consists of homogeneously distributed ultrasmall (≈1 nm) amorphous calcium phosphate clusters. In vitro study shows the strong affinity of CaP‐PILP to osteoporotic bone, which can be uniformly distributed throughout the bone tissue to significantly increase the bone density. In vivo experiments show that the repaired bones exhibit satisfactory mechanical performance comparable with normal ones, following a promising treatment of osteoporosis by using CaP‐PILP. The discovery provides insight into the structure and property of biological nanocluster materials and their potential for hard tissue repair.

Bone is a hierarchical hard tissue that consists of oriented crystals of hydroxyapatite (HAP, Ca_10_(PO_4_)_6_(OH)_2_) embedded within type I collagen fibrils.^1^, ^2^ The 2–4 nm thick HAP crystalline platelets in bone significantly enhance the stiffness and toughness of the collagen fibrils by dissipating the tension loaded on the structure.^3^, ^4^, ^5^ Undernutrition and protein deficiency can cause a reduced bone mineral density, which increases the risk of osteoporosis.^6^, ^7^ Osteoporosis is a worldwide bone disease, characterized by a lack of HAP in bone and an associated increased risk of fracture.^8^, ^9^ The current clinical treatments of osteoporosis largely rely on drugs, including vitamin D,^10^, ^11^ bisphosphonate,^12^, ^13^ and calcium supplements.^14^ However, as these treatments cannot return osteoporotic bone to its healthy state, osteoporosis is now generally accepted as an incurable chronic disease.^15^ Up to now, autogenous bone grafts have been considered the “gold standard” for bone replacement, but have limited availability and potential compatibility issues.^16^, ^17^ Hence, many bone‐substitute materials such as bone cement, mesoporous scaffolds, synthetic composites, and hydrogels have been explored for bone regeneration.^18^, ^19^, ^20^, ^21^, ^22^ However, these repair materials are not able to regenerate osteoporotic bone by remineralizing bone collagen fibrils. Here, we propose that osteoporotic bone can be recovered by remineralizing the collagen fibrils in the affected bone. Although a number of calcium phosphate (CaP)‐based biomedical nanomaterials have been developed and successfully used for repairing bone defects,^23^, ^24^, ^25^ remineralizing the collagen fibrils in bone in vivo remains a great challenge. This limitation can be understood by taking a close look at the collagen fibril structure. The collagen fibril is an assembly of quasi‐hexagonally packed, twisted collagen triple‐helix molecules^26^ that contain only ≈1.8–4 nm sized tortuous subchannels.^27^ Such a structure is inaccessible to most CaP nanomaterials and thereby makes the recovery of the affected bone difficult.

The polymer‐induced liquid‐precursor (PILP) was first reported for calcium carbonate^28^ and was then extended to calcium phosphate.^29^, ^30^, ^31^ PILP is a liquid‐like mineral precursor stabilized by charged polymers, such as polyacrylic acid (PAA),^31^ poly(allylamine hydrochloride) (PAH),^32^ or polyaspartic acid (PASP),^28^ that forms thin films on flat substrates and can infiltrate into nanopores.^28^, ^33^, ^34^ In vitro experiments have shown that the calcium phosphate PILP is able to infiltrate into collagen fibrils and form oriented intrafibrillar HAP crystallites, with a diffraction pattern indistinguishable from that of the mineralized collagen fibrils in bone.^31^, ^35^ Despite the unique liquid‐like properties of PILP and its possible vital role in the biomineralization processes,^36^ its microstructure is still under debate. While early studies suggest that PILP appears as a dense liquid phase,^28^, ^37^ cryo‐transmission electron microscopy (cryoTEM) observations showed only amorphous calcium phosphate (ACP) nanoclusters in the early stage of in vitro collagen remineralization experiments, where the calcium phosphate PILP is supposed to form.^33^, ^35^ Similarly, a recent cryoTEM study using a calcium carbonate system indicated that PILP is actually a polymer‐driven assembly of nanoclusters.^34^ Although calcium phosphate PILP displays a promising ability to remineralize collagen fibrils, it has not yet been applied in biomedical bone engineering. A major challenge is that the calcium phosphate PILP is generally synthesized at low Ca^2+^/PO_4_^3−^ concentrations,^31^ which are insufficient to provide the mineral mass required for the recovery of demineralized bone at the macroscopic scale.

In the present work, we demonstrate that the full recovery of osteoporotic bone can be achieved using a free‐flowing calcium phosphate polymer‐induced liquid‐precursor (CaP‐PILP) material. By combining two biocompatible polymeric additives, PAA and PASP, CaP‐PILP is stabilized on a large scale and at a high Ca^2+^/PO_4_^3−^ concentration. In contrast to previous CaP materials for bone repair, this CaP‐PILP material has excellent bone inductivity, which uniquely allows the intrafibrillar mineralization of collagen fibrils. This is directly related to its microstructure, which contains a high density of uniform‐sized (≈1 nm) ACP nanoclusters. Both in vitro and in vivo experiments provide the first proof that the structural and mechanical properties of osteoporotic bone can be recovered to those of healthy bone by treatment with CaP‐PILP.

Two biocompatible, negatively charged polymers, PAA and PASP, were used in the synthesis of our CaP‐PILP material. PAA with sufficient molecular weight is able to stabilize the PILP phase of CaP.^38^ However, this polymer also causes precipitates to form when mixed with high concentrations of Ca^2+^. To generate a stable CaP‐PILP at a high Ca^2+^ without precipitation, we used PASP to bind Ca^2+^ as a competitor to PAA. In a typical procedure, 2.0 mL of a 0.1 m CaCl_2_ solution was mixed with 0.2 mL of a 0.3 g mL^−1^ PASP solution to obtain solution A, while 2.0 mL of a 0.1 m Na_2_HPO_4_ solution was mixed with 0.4 mL of a solution containing 0.3 g mL^−1^ PAA to obtain solution B. Then, 2.4 mL of solution B was slowly injected into 2.2 mL of solution A with vigorous stirring. The negatively charged carboxylate groups on PAA/PASP chains can bind with Ca^2+^ and prevent it from precipitating immediately with HPO_4_^2−^, so that the PILP phase can be formed. The PILP phase forms with relatively low concentration of charged polymers (<20 mg L^−1^) and Ca^2+^ (≤5 × 10^−3^ m).^31^, ^35^ However, in this work we aim to form a high concentration of CaP‐PILP so that it can sufficiently support the repair of osteoporotic bones, therefore a high concentration of PAA/PASP (26.1 and 13.0 mg mL^−1^) was used, with the maximal amount of Ca^2+^ that can be chelated by the PAA/PASP, which is 43.5 × 10^−3^ m. The resulting material is transparent and viscous but still free flowing (**Figure** **1**a; Movie S1, Supporting Information). Cryogenic electron tomography (cryoET) showed that the resulting material is densely loaded with uniform‐sized, separate, and homogeneously distributed nanoclusters, indicating the formation of CaP‐PILP (Figure 1b; Movie S2, Supporting Information). In the PILP process, PAA and PASP are used to stabilize and form an amorphous precursor that is sufficiently hydrated to be a liquid phase. The close‐to‐focus cryoTEM images (defocus = −1 µm) showed that the clusters are ≈1 nm in size (inset 1 of Figure 1b).^5^, ^39^ Selected area electron diffraction (SAED, inset 2 of Figure 1b) showed a broad diffraction band, while powder X‐ray diffraction (pXRD) showed a broad peak at ≈2θ = 30° (Figure S1a, Supporting Information); both results indicate that the clusters are ACP. This assignment was further confirmed by Fourier transform infrared (FT‐IR) spectroscopy, which revealed two wide bands typical of phosphate stretching (*v*_3_) at 1055 cm^−1^ and phosphate bending (*v*_4_) at 560 cm^−1^ (Figure S1b, Supporting Information). The dynamic mechanical properties of CaP‐PILP were examined by frequency‐dependent oscillatory shear rheology (Figure 1c). The measurements revealed a dynamic storage modulus (*G*′) that was slightly lower than the loss modulus (*G*″), confirming that CaP‐PILP is a fluid despite its very high viscosity. The strain‐dependent oscillatory rheology of CaP‐PILP exhibited a broad linear viscoelastic region, indicating that this material has a wide processing range within the strain domain of 0.1%–100%. Taken together, the results indicate that CaP‐PILP is a viscous, transparent, liquid‐like precursor phase with a high density of uniform‐sized ACP clusters.

**Figure 1:**
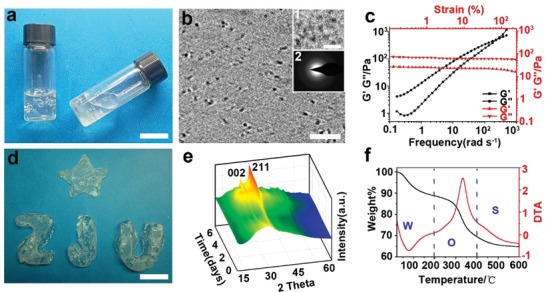
Preparation, fluidity, and characterization of the CaP‐PILP. a) Photograph of CaP‐PILP, indicating that the material is viscous but still free flowing. b) Cross‐section through the 3D reconstruction of a cryoET image of CaP‐PILP, showing that the nanoclusters are homogeneously distributed and separated. Zoomed‐in and close‐to‐focus cryoTEM images showing that the size of the clusters is ≈1 nm (inset 1), while the SAED pattern shows that the clusters are amorphous (inset 2). c) Rheological characterization of CaP‐PILP, showing the frequency‐dependent (at a strain of 1%, black lines) and strain‐dependent (ω = 10 rad s^−1^, red lines) oscillatory shear rheology. d) Solidified CaP‐PILP with different shapes. e) 3D pXRD patterns of the solidified CaP‐PILP during solidification. f) TG (black line)/DTA (red line) curves of the solidified CaP‐PILP. The weight loss at 200 °C is attributed to the loss of water, and the dominant weight loss occurs between 200 and 400 °C, corresponding to the decomposition of organics in the solidified CaP‐PILP. W: water, O: organics, S: the solidified CaP‐PILP. Scale bars: a,d) 2 cm, b) 10 nm, 5 nm (inset 1 in (b)).

The liquid‐like CaP‐PILP can be solidified after being injected into moulds kept at 37 °C for 7 d (Figure 1d). Conventional transmission electron microscopy (TEM) showed that the amorphous phase transforms into nanorod/nanoplate‐like structures with a length of ≈20 nm (Figure S2a, Supporting Information). SAED (Figure S2b, Supporting Information) and pXRD (Figure 1e) provided evidence for an amorphous‐to‐crystal transition, showing an increase in the reflections corresponding to both the (002) and (211) planes of HAP over time. The formation of HAP was further confirmed by the appearance of phosphate *v*_4_ bending vibrations at 565 and 600 cm^−1^ at the expense of the broad band at 560 cm^−1^ (Figure S2c, Supporting Information).^40^ The thermogravimetric and differential thermal analysis (TG/DTA) measurements (Figure 1f) revealed an endothermic peak between 20 and 200 °C and an exothermic peak between 200 and 400 °C, which are assigned to the loss of water and the decomposition of organics, respectively. The TG curves showed that the solidified material is composed of 69.4 wt% mineral, 19.3 wt% organics, and 11.3 wt% water. The solidification and crystallization of CaP‐PILP is related to the conversion of the ≈1 nm clusters, which is similar to the 0.7–1.0 nm sized “Posner's clusters.”^39^, ^41^ Posner's clusters act as the basic building blocks to generate larger ACP nanoparticles by cluster‐cluster complex aggregation.^5^ By taking up the extra‐OH groups and calcium ions into the voids within the ACP precursors, HAP can be further formed.^42^, ^43^ Generally, the solidification and crystallization of these clusters to HAP is fast and occurs within hours.^5^ However, those processes are extended to days in our CaP‐PILP due to the stabilization effect from PAA and PASP.

The CaP‐PILP was then used for the remineralization of type I collagen fibrils. The native collagen fibrils (self‐assembled from rat tail type I collagen) display periodic gap and overlap regions (**Figure** **2**a). The TEM grids coated with the collagen fibrils were then floated at 37 °C oven the CaP‐PILP as well as a suspension of commercial HAP nanocrystals. After 7 d of contact with the commercial HAP nanocrystals, the fibrils were barely mineralized, and HAP nanocrystals were observed only around the collagen fibrils (Figure 2b). In contrast, the CaP‐PILP‐treated fibrils showed increased levels of mineralization with time (Figure 2c–e), while SAED confirmed that HAP is the final mineral product (insets in Figure 2c–e). The intrafibrillar mineralization of collagen was demonstrated using 3D super‐resolution stochastic optical reconstruction microscopy (STORM) (Figure 2f–j). For this experiment, the collagen fibrils were labeled before mineralization with the red‐emitting fluorescent reagent cy3B. After 7 d, the mineralized collagen fibrils were stained with 10.0 × 10^−6^ m calcein to label the newly generated HAP nanocrystals. The results showed that crystals form within the collagen fibrils (Figure 2j), and the degree of mineralization in the collagen fibrils is approximately 95%.

**Figure 2:**
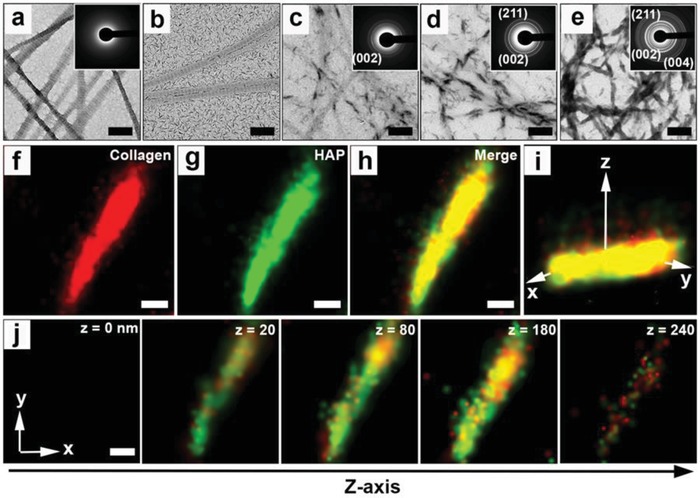
Mineralization process of collagen fibrils. a) TEM image and SAED pattern (inset) of the native collagen fibrils. b) Failed mineralization of collagen fibrils after exposure to HAP for 7 d at 37 °C. c–e) TEM images and SAED patterns (insets) of the mineralization process of collagen fibrils using the CaP‐PILP at different time points at 37 °C: c) 3 d, d) 5 d, and e) 7 d. SAED patterns showing diffraction rings corresponding to the (002), (211), and (004) crystallographic planes of HAP (insets in (c–e)). f–h) The *xy* projections of STORM images of mineralized collagen fibrils. f) The collagen fibrils were labeled with cy3B, emitting red fluorescence, while g) HAP was labeled with calcein, emitting green fluorescence. i) 3D STORM image of the mineralized collagen fibrils shown in inset (h). j) Z‐slice of STORM images of the mineralized collagen fibrils, indicating that the HAP is on/in the collagen fibrils. Scale bars: a,b,f–j) 200 nm and c–e) 600 nm.

To exclude possible endotoxin contamination, suspensions of commercial ACP nanoparticles (ACP group, size of ≈80 nm, Figure S3a, Supporting Information), HAP nanoparticles (HAP group, ≈150 × 30 × 1.5 nm, Figure S3b, Supporting Information), or CaP‐PILP were incubated with RAW264.7 cells and assessed for the secretion of the inflammatory cytokine IL‐6, respectively, which is a sensitive readout for the presence of endotoxins.^44^ The IL‐6 release of CaP‐PILP was similar to that of the ACP, HAP, and control groups after culturing for 1, 6, and 24 h, indicating that CaP‐PILP did not promote the secretion of IL‐6 compared with the ACP, HAP, and control groups (Figure S4, Supporting Information). To investigate the biocompatibility and osteoinductive capacity of CaP‐PILP, bone marrow‐derived mesenchymal stem cells (MSCs) were cultured with CaP‐PILP using ACP group, HAP group, and osteogenic medium only (blank group) as controls. In terms of cell differentiation, the expression of alkaline phosphatase (ALP) in MSCs cultured with the ACP, HAP, and CaP‐PILP groups all increased after 7 d compared with that of the blank group (Figure S5a–d,m, Supporting Information). The ALP activity of the CaP‐PILP group is similar to that of the HAP group, slightly higher than that of the ACP group, and ≈2.4 times higher than that of the blank group, revealing that CaP‐PILP can promote the differentiation of MSCs (Figure S5m, Supporting Information). Another biochemical marker of in vitro osteogenic differentiation, calcium deposition,^45^ was also investigated for the MSCs after culturing with osteogenic medium for 14 d (Figure S5e–h,n, Supporting Information). We observed that the calcium deposition increased for the ACP, HAP, and CaP‐PILP groups compared with that of the blank group (Figure S5e–h, Supporting Information). The quantitative analysis showed that the optical density (OD) value of the CaP‐PILP group was ≈2.0, 1.5, and 20.0 times that of the HAP, ACP, and blank groups, respectively (Figure S5n, Supporting Information). However, without MSCs, calcium deposition of the four groups with osteogenic medium was relatively low, and the OD values of the blank, ACP, HAP, and CaP‐PILP groups were 4.6 × 10^−2^, 5.1 × 10^−2^, 4.7 × 10^−2^, and 6.2 × 10^−2^, respectively (Figure S5i–l,o, Supporting Information). In general, the results indicated that CaP‐PILP provides a suitable physicochemical and biological microenvironment for the differentiation of MSCs, which is essential for in vivo osteoporotic bone recovery.

The affinity of CaP‐PILP for bone was studied by measuring the permeability of a rhodamine B‐containing droplet of CaP‐PILP into osteoporotic bone, which simulates the in vivo infiltration of CaP‐PILP to osteoporotic bone (**Figure** **3**a–d). After placing the purple CaP‐PILP droplet on the milky white osteoporotic bone for 30 s, the droplet extended on the surface of the osteoporotic bone (Figure 3b). After 2 h, the purple color was uniformly distributed throughout the osteoporotic bone, indicating excellent permeability (Figure 3c,d). After treatment with CaP‐PILP for 1 d, ACP was observed surrounding and entering the collagen fibrils (Figure 3f), and remineralization of the collagen fibrils could be observed after 7 d, The SAED patterns confirmed that the mineral phase is HAP (Figure 3g). In contrast, without CaP‐PILP treatment, demineralized collagen fibrils were observed in the osteoporotic bone (Figure 3e). We then investigated the in vitro osteoporotic bone recovery ability of CaP‐PILP by injecting a suspension of HAP particles or CaP‐PILP into osteoporotic bones. After incubation at 37 °C for 2 weeks, the samples were analyzed by micro‐computed tomography (micro‐CT), with the native, untreated osteoporotic bone, and healthy bone used as comparisons (Figure 3h–k). The HAP group showed little bone recovery (Figure 3i) and was similar to osteoporotic bone (Figure 3h). In contrast, the CaP‐PILP group showed a clear recovery of osteoporotic bone (Figure 3j), with a result that was comparable to healthy bone (Figure 3k). To quantify the amount of newly recovered bone, histomorphometry analysis was performed to obtain the bone volume/tissue volume ratio (BV/TV). The BV/TV values of the osteoporotic bone, HAP recovered bone, CaP‐PILP recovered bone, and healthy bone were 0.13, 0.19, 0.45, and 0.47, respectively, indicating the excellent performance of CaP‐PILP in recovering osteoporotic bone (Figure 3l). The scanning electron microscopy (SEM) images further supported the micro‐CT results, showing the high porosity of the HAP (Figure S6a, Supporting Information) and osteoporotic bone groups (Figure 3m) compared with that of the CaP‐PILP (Figure 3q) and healthy groups (Figure S6b, Supporting Information). Scanning transmission electron microscopy (STEM) showed that osteoporotic bone is deficient in calcium and phosphate (Figure 3n–p), and the SAED patterns confirmed that there is little mineral present (inset in Figure 3n). However, abundant calcium and phosphate atoms were in the CaP‐PILP recovered bone (Figure 3r–t). The diffraction patterns of HAP for the CaP‐PILP recovered bone, however, showed (002) diffraction arcs following the long axis of the collagen fibrils (inset in Figure 3r). These results demonstrated that CaP‐PILP can effectively recover osteoporotic bone in vitro.

**Figure 3:**
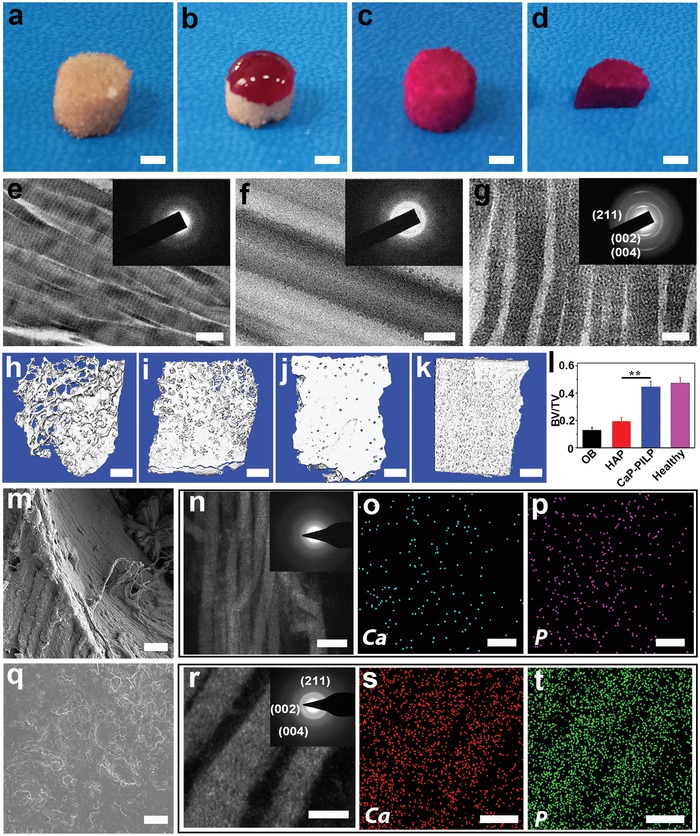
Affinity of CaP‐PILP to osteoporotic bone and in vitro results of osteoporotic bone and recovered bone. a–d) Affinity of CaP‐PILP to osteoporotic bone. a) Osteoporotic bone. b–d) The affinity of CaP‐PILP was measured by placing drops of the purple CaP‐PILP staining solution onto the osteoporotic bone at 37 °C for b) 30 s and c) 2 h. d) The section of the stained osteoporotic bone indicated that CaP‐PILP was uniformly distributed throughout the osteoporotic bone after 2 h. e–g) TEM images and SAED patterns of e) osteoporotic bone (inset in (e)), f) CaP‐PILP treated bone for 1 d (inset in (f)), and g) 7 d (inset in (g)). h–k) Representative 3D micro‐CT of h) osteoporotic bone, i) HAP recovered bone, j) CaP‐PILP recovered bone, and k) healthy bone. l) The quantitative results of the 3D micro‐CT analysis expressed as BV/TV. OB: osteoporotic bone. HAP: HAP‐recovered bone. CaP‐PILP: CaP‐PILP recovered bone. Healthy: sham‐operation bone. *n* = 3, ^**^p < 0.01. m) SEM image of osteoporotic bone. n–p) Representative STEM image (n), SAED pattern (inset in (n)) and the corresponding element mapping (o–p) of osteoporotic bone. Calcium (o) and phosphate (p) atoms were labeled by blue and purple, respectively. q) SEM image of CaP‐PILP recovered bone. r–t) Representative STEM image (r), SAED pattern (inset in (r)) and the corresponding element mapping (s–t) of CaP‐PILP recovered bone. Calcium (s) and phosphate (t) atoms were labeled by red and green, respectively. Scale bars: a–d) 100 µm, e–g,r–t) 100 nm, h–k) 0.5 mm, m,q) 10 µm, n–p) 200 nm.

Subsequently, the in vivo osteoporotic bone recovery capability of CaP‐PILP was evaluated in ovariectomized osteoporotic mouse tibia using a percutaneous mini‐invasive injection syringe at 4, 8, and 12 weeks (**Figure** **4**a). To determine the location of the injected CaP‐PILP, in vivo imaging was performed on living osteoporotic mice to locate the fluorescence signals from calcein‐stained CaP‐PILP (Figure S7, Supporting Information). After injecting the calcein‐stained CaP‐PILP droplet into the osteoporotic mouse tibia for 30 min, the green fluorescence signal from CaP‐PILP extended into the osteoporotic bone (Figure S7a, Supporting Information). After 2 h, the green color infiltrated throughout the tibia, indicating that CaP‐PILP has been nicely distributed in the tissue (Figure S7b, Supporting Information). The bone loss results from oestrogen deficiency due to enhanced bone resorption and impaired osteoblast function.^46^ In the experiments, the control group was subjected to a bilateral ovariectomy, which limited the secretion of oestrogen, resulting in osteoporosis. The control group cannot heal naturally during the lifetime of the mice because the osteoporotic bone lacked mineral supply. Representative 2D and 3D micro‐CT images of the osteoporotic bone, phosphate‐buffered saline (PBS), CaP‐PILP, and healthy bone (sham‐operation) groups at postoperative weeks 0, 4, 8, and 12 are provided in Figure 4b–i and Figures S8–S10 in the Supporting Information. In the osteoporotic bone and PBS groups, hardly any new bone formation occurred over time (Figures S8–S10, Supporting Information). In contrast, after 4 weeks, new bone formation was significantly increased in the CaP‐PILP group (Figure 4c,g). After 8 weeks, the healing status of the CaP‐PILP group (Figure 4d,h) was already comparable with that of the healthy bone group (Figures S8c,f, S9c,f, and S10c,f, Supporting Information). No further growth of new bone tissue was detected at postoperative week 12 (Figure 4e,i), indicating that in the CaP‐PILP group, the bone recovery reached its summit after 8 weeks. Haematoxylin and eosin (H&E) staining also demonstrated that the CaP‐PILP group showed abundant newly formed bone tissue after 8 and 12 weeks (Figure 4l,m) and was nearly comparable with the healthy bone group (Figures S8i, S9i, and S10i, Supporting Information), while scare newly formed bone was detected in the osteoporotic bone and PBS groups (Figures S8g,h, S9g,h, and S10g,h, Supporting Information). The BV/TV, trabecular number (Tb.N) and trabecular separation (Tb.Sp) of the four groups were analyzed to quantify the amounts of osteoporotic bone and newly formed bone (**Figure** **5**a–c) and were shown to remain constant for the osteoporotic bone, PBS, and healthy bone groups after 4, 8, and 12 weeks. The BV/TV and Tb.N of the CaP‐PILP group, however, increased by factors of approximately 2.6 or 1.3, respectively, after 8 weeks, while the Tb.Sp decreased. The values were all comparable with those of the healthy bone group, indicating that CaP‐PILP remarkably promotes new bone formation in osteoporotic regions. Similar to the in vitro experiments, elemental mapping and SAED revealed that the osteoporotic bone and PBS recovered bone showed a strongly reduced mineral content (Figure S11a,b, Supporting Information), while the CaP‐PILP recovered bone showed the formation of HAP crystals with their *c*‐axis aligned along the collagen fibrils, similar to that in healthy bone (Figure S11c,d, Supporting Information). These results confirmed that treatment with CaP‐PILP is a promising method for rapid osteoporotic bone recovery in vivo.

**Figure 4:**
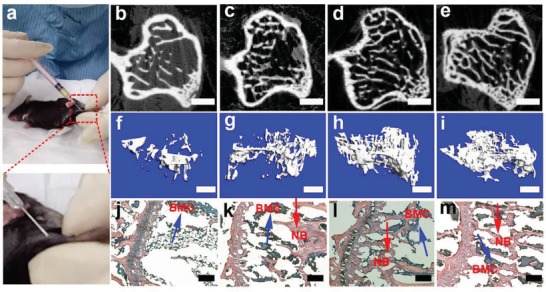
Schematic of the percutaneous mini‐invasive injection and in vivo results of CaP‐PILP‐recovered bone after 0, 4, 8, and 12 weeks. a) Thirty 30 µL of prepared CaP‐PILP was percutaneously mini‐invasively injected into the mouse osteoporotic tibia. b–m) Representative 2D, 3D micro‐CT, and H&E staining of CaP‐PILP‐recovered bone at b,f,j) 0 weeks, c,g,k) 4 weeks, d,h,l) 8 weeks, and e,i,m) 12 weeks. NB (red arrows): new bone. BMC (blue arrows): bone marrow cells. Scale bars: b–e) 100 µm; f–i) 300 µm; and j–m) 200 µm.

**Figure 5:**
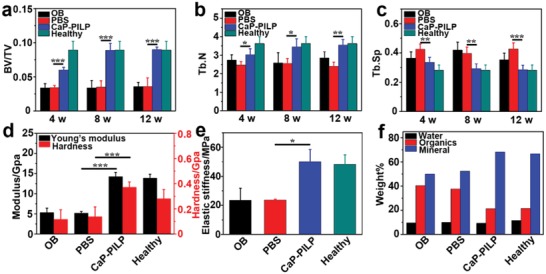
Quantitative results of micro‐CT analysis, mechanical testing, and TG analysis of osteoporotic bone, recovered bone, and healthy bone. Quantitative results of micro‐CT analysis expressed as a) BV/TV, b) Tb.N, and c) Tb.Sp. d) Young's modulus and hardness, e) stiffness, and f) TG analysis of the four groups. The results showed that the inorganic ratio of CaP‐PILP‐recovered bone is similar to that of healthy bone, while the inorganic ratio of PBS‐recovered bone is similar to that of osteoporotic bone. OB: osteoporotic bone, PBS: PBS‐recovered bone, CaP‐PILP: CaP‐PILP‐recovered bone, Healthy: sham‐operation bone. a–c) *n* = 5, *p < 0.05, d) *n* = 6, ***p < 0.001, e) *n* = 3, *p < 0.05.

The mechanical properties of the osteoporotic bone and recovered bone were also tested (Figure 5d–e). The results showed that the hardness values of the osteoporotic bone, PBS recovered bone, CaP‐PILP treated bone, and healthy bone were 116.5, 137.7, 371.8, and 280.5 MPa, respectively, while the Young's moduli were 5.3, 5.2, 14.3, and 13.9 GPa, respectively (Figure 5d). The recorded compressive stress–strain curve can be divided into three main regions: linear elastic, plateau, and densification. For these four groups, the stiffness of the materials was determined from the maximum value of the stress–strain slope in the linear elastic region.^47^ The compressive stress–strain measurements indicated that the stiffness values of the osteoporotic bone, PBS recovered bone, CaP‐PILP recovered bone, and healthy bone were 23.4, 23.6, 50.2, and 48.3 MPa, respectively (Figure 5e). These results demonstrate that our CaP‐PILP can effectively enhance the mechanical performance of osteoporotic bone and that the recovered zones displayed a similar (even higher) stiffness and hardness to those of healthy bone, which makes CaP‐PILP an excellent candidate for osteoporotic bone recovery. The TG curves showed that the CaP‐PILP recovered bone was composed of 68.3 wt% mineral, a value that was very similar to what was found for healthy bone (66.8 wt% mineral), while the mineral ratio in the osteoporotic bone and PBS groups was only ≈50.0 wt% (Figure 5f).

In this work, the bone‐inductive CaP‐PILP is synthesized and used for osteoporotic bone recovery as an alternative to traditional osteoporotic bone treatment methods. The resulting CaP‐PILP can penetrate into osteoporotic bone tissue to induce the intrafibrillar mineralization of collagen fibrils with HAP, a key aspect in effectively recovering osteoporotic bone tissue. The recovered bone displays good mechanical performance and is comparable with healthy bone. The fluidity of CaP‐PILP allows for the minimally‐invasive injection recovery of osteoporotic bone, without the need for surgical incision in clinical applications. More generally and fundamentally, our results provide the first proof that the structure and mechanical performance of osteoporotic bone can be recovered to their healthy state by treatment with CaP‐PILP.

## Experimental Section

*Preparation of CaP‐PILP*: In a typical procedure, 2.0 mL of a 0.1 m CaCl_2_ solution was first mixed with 0.2 mL of a 0.3 g mL^−1^ PASP (*M*_w_ = 9–11 kDa) solution to obtain solution A, while 2.0 mL of a 0.1 m Na_2_HPO_4_ solution was mixed with 0.4 mL of a solution containing 0.3 g mL^−1^ PAA (*M*_w_ = 450 kDa) to obtain solution B. Then, 2.4 mL of solution B was slowly injected into 2.2 mL of solution A with vigorous stirring, and the pH value was adjusted to 7.4 with NaOH solution.

*CryoTEM of CaP‐PILP*: CryoTEM Au grids (R2/2 Quantifoil Jena Grids) were treated by glow discharge for 40 s to increase their hydrophilicity. Three microliter of CaP‐PILP was applied on the grid, and then the grid was blotted for 3 s, relaxed for 60 s to allow the formation of a thin liquid layer, and vitrified by plunging into liquid ethane at liquid nitrogen temperature. CryoTEM imaging was performed under an ≈1 µm defocus on an FEI‐Titan TEM equipped with a field emission gun operating at 300 kV. The images were recorded using a 2k  ×  2k Gatan CCD camera equipped with a postcolumn Gatan energy filter (GIF), with an electron dose of 16 e Å^−2^ per image. A cryogenic tomography tilt series was recorded by tilting the holder from −65 to +65 degrees using the Saxton tilt increment scheme (87 images were taken in total).^48^ The images were recorded under an ≈3 µm defocus, with an electron dose of 2 e Å^−2^ per image.

*Rheological Test*: The rheology experiments were performed on an Anton Paar rheometer at 25 °C. CaP‐PILP was prepared and gently placed in the middle of a 15 mm diameter parallel plate with a proper gap. Dynamic oscillatory frequency sweep measurements were conducted at a 1% strain amplitude. To prevent evaporation, a lid was prepared on the top.

*Pro‐Inflammatory Cytokines (IL‐6)*: To test the inflammatory response of CaP‐PILP, RAW264.7 cells were cultured with CaP‐PILP, a suspension of ACP particles, a suspension of HAP particles, and medium only at 37 °C for 1, 6, and 24 h, respectively. The concentration of the cytokines IL‐6 was measured by ELISA using antibodies obtained from Biolegend according to the manufacturer's instructions (R&D Systems).

*Osteogenic Differentiation*: Four types of media were prepared and coated onto 24‐well Petri dishes: 50 µg CaP‐PILP, commercial ACP particles, HAP particles, and a blank. All the groups were sterilized overnight under ultraviolet germicidal lamps. The osteogenic medium was composed of 10^−8^ m dexamethasone, 50 µg mL^−1^ ascorbic acid, 10 × 10^−3^ m β‐glycerol phosphate, 10% fetal bovine serum (FBS), and high‐glucose Dulbecco's modified Eagle's medium (DMEM). Then, MSCs were seeded in the above 24‐well Petri dishes at a density of 1 × 10^4^ cells/well. The media changed every other day, and the MSCs in the four groups were incubated at 37 °C in a humidified atmosphere containing 5% CO_2_. After the MSCs were cultured for 1 week in osteogenic medium, the ALP activity was examined using a commercial detection kit (Beyotime, C3206). The cell nuclei were stained with 4′, 6‐diamidino‐2‐phenylindole (DAPI) to count the total cell number and calculate the ALP staining positive rate of the MSCs. The calcium deposits formed were also stained by the MSCs with Alizarin Red S (ARS) after culturing in osteogenic medium for 14 d. To further quantify the results of the ARS staining, the stained nodules were solubilized with 5% sodium dodecyl sulfate (SDS) in 0.5 m HCl for 30 min at room temperature. Finally, the OD value of the solution was measured at a wavelength of 405 nm.

*Self‐Assembly of Collagen Fibrils on the TEM Grids and Laser Confocal Culture Dish (LCCD) and Collagen Mineralization*: A 3 mg mL^−1^ stock solution of type I collagen was purchased from Gibco‐Invitrogen. The assembly solutions contained 50 × 10^−3^ m glycine and 200 × 10^−3^ m KCl, and the pH was adjusted to 9 using NaOH solution. An 8.33 µL volume of the collagen stock solution was added dropwise into 0.5 mL assembly solution and incubated for 20 min at 37 °C. 3 µL of the incubated collagen solution was placed on a nickel TEM grid for 12 h and then rinsed with deionized water. For the LCCD samples, 100 µL collagen solution (50 µg mL^−1^) was placed dropwise over an aminopropyltriethoxysilane (APTES)‐modified LCCD glass substrate, incubated at a constant temperature of 37 °C for 12 h and washed with deionized water. Then, the collagen fibrils were further cross‐linked with 0.05% glutaraldehyde for 4 h. TEM grids loaded with collagen fibrils were floated on the CaP‐PILP for mineralization. The mineralization degree of the collagen fibrils was quantified using ImageJ based on the method used in the previous work.^49^ Briefly, the pixel intensities of the mineralized collagen fibrils and nonmineralized collagen fibrils in the TEM images was different. The nonmineralized region contains light atoms (C, H, and N), while the mineralized portion contains extra heavier atoms (Ca and P). As a result, the mineralized region has a lower pixel intensity comparing with the nonmineralized region, and the areas of the mineralized region (*S*1) and nonmineralized region (*S*2) can be obtained by segmenting the image based on pixel intensities. The mineralization degree (*m.d*.) is calculated by (six TEM images were examined to obtain the mean mineralization degree)

$$m.d.\;=\frac{S1}{S1+S2}$$

*3D STORM Imaging*: The collagen fibrils were labeled with a fluorescent reagent by immunofluorescence staining. CaP‐PILP was incubated with blocking buffer (Beyotime, China, Product Code: P0023B) for 1 h at 37 °C. After washing three times, the samples were incubated with Cy3B‐conjugated secondary antibodies for 2 h. After that, 1 mL of CaP‐PILP was placed dropwise onto the LCCD, which was loaded with immunofluorescence‐stained fibrils. The material was incubated at 37 °C for 6 h and rinsed with deionized water three times. Then, the mineralized collagen fibrils were labeled with 10 × 10^−6^ m calcein for 20 min and rinsed with deionized water three times. All STORM imaging experiments were performed on a Nikon Ti‐E inverted optical microscope, the movies and images were analyzed by Nikon NIS‐Elements AR software.

*In Vitro Recovery of Osteoporotic Bone*: In vitro experiments were used to detect the mineralization properties of CaP‐PILP for collagen fibrils in osteoporotic bones without cells or vessels. Female Sprague Dawley osteoporotic bones and healthy bones were kindly provided by the Sir Run Run Shaw Hospital Affiliated with the Medical College of Zhejiang University, and the use of animal tissues for the in vitro study was approved by the guidelines on the care and use of animals for scientific purposes issued by the National Institutes of Health (NIH) and Zhejiang University. First, models with ovariectomized‐induced 8‐week‐old female Sprague Dawley (body weight, 290–330 g) osteoporotic rats were created. Then the healthy rats and osteoporotic rats were scarified to obtain the femurs. After that, the healthy femurs and osteoporotic femurs were cut into slices and dried in an oven at 37 °C for seven days prior to use. CaP‐PILP was synthesized by the above method, and the HAP was made by suspending commercial HAP particles into a PBS solution. CaP‐PILP or HAP particles were injected into the osteoporotic bones, and then the bones were placed in a water bath at 37 °C for 14 d. After that, the bones were dried at room temperature before further experiments.

*In Vivo Recovery of Osteoporotic Bone*: All animal experiments were performed at the Sir Run Run Shaw Hospital Affiliated with the Medical College of Zhejiang University. All handling and care of the animals were carried out according to the guidelines on the care and use of animals for scientific purposes issued by the NIH and Zhejiang University. First, models with ovariectomized‐induced osteoporotic bone were created. All mice in this model were deprived of any food for 6 h before being anaesthetized. Each mouse in this model was given a general anesthetic of 50 mg kg^−1^ pentobarbital sodium by intraperitoneal injection and then fixed in the prone position. The psoas muscles were cut along the linea scapularis subcostals at the two sides to expose the ovaries and uterine horns under the kidneys, and then ligature was conducted. Subsequently, the uterine horns were cut, the ovaries were completely extracted, the incision was sewn closed layer by layer, and the model creation surgery was complete. The removed tissue was examined to ensure the completeness of the surgery, and the ovaries were confirmed by histological determination. In the sham‐operation mice, the incisions were made without resection. Briefly, 65 healthy 8‐week‐old female C57BL/6 mice (body weight, 20–25 g) were used in this study. 50 mice were randomly selected for the ovariectomized groups, and the rest (15 mice) were used as the healthy group (sham operation). The ovariectomized mice were randomly divided into three groups: osteoporotic bone (no intervention), PBS, and CaP‐PILP (*n* = 10) groups. The different administrations began at the 6th week after oophorectomy. All the materials were filtered through 0.22 µm Millipore films prior to use. Then, 30 µL of the prepared CaP‐PILP or PBS was percutaneously mini‐invasively injected into the osteoporotic tibia after anaesthetization (30 µL is the maximum injection). After that, all the mice remained in good health and did not show any wound symptoms throughout four, eight, and twelve weeks of experiments. All mice were sacrificed after 0, 4, 8, and 12 weeks according to the animal ethics regulations. The uteri were isolated and weighed to confirm the effects of the ovariectomy, and the tibia specimens were harvested and fixed in 4% (w/v) paraformaldehyde for further observation.

*Micro‐CT Scanning*: A high‐resolution micro‐CT (Skyscan 1072; Skyscan, Aartselaar, Belgium) scanner was used to analyze the fixed tibia, operating at a voltage of 80 kV and a current of 80 µA. The micro‐CT scanner's auxiliary software was used to make a 3D reconstruction from the sequential scans. The quantitative results of the micro‐CT analysis were measured using the CTAn program.

*Histological Procedure*: The specimens were fixed with 4% paraformaldehyde and decalcified in 10% ethylenediaminetetraacetic acid for 4 weeks at pH 7.1 and 4 °C. The samples were then embedded in paraffin, and serial sections with a 5 µm thickness were prepared. Three randomly selected sections from each implant were stained with H&E, and the sections were observed by optical microscopy (Nanozoomer, 2.0RS, Hamamatsu, Japan).

*Evaluation of Mechanical Properties*: After the mice were sacrificed, the tibias were removed intact from the surrounding bone. The Young's modulus and hardness of the CaP‐PILP‐recovered bone and PBS‐recovered bone were compared to the properties of the osteoporotic bone and healthy bone. A nanoindentation test using the Berkovich tip was used to analyze the modulus and hardness of each section (G200, Agilent Technologies, CA, USA). The data were recorded and managed by Testworks 4 software (MTS System Corporation, Eden Prairie, MN, USA), which calculated the modulus and hardness. The ultimate compressive stress–strain was determined by using a computer‐controlled test machine (Z2.5, Zwick/Roell, Ulm, Germany). Each tibia was cut into a 0.5 cm portion to measure the mechanical properties. The samples were compressed to failure at a rate of 1 mm min^−1^. Three replicates for each group were tested. The compressive stress–strain curve can be divided into three main regions: linear elastic, plateau, and densification. To calculate the stiffness, the initial nonlinear behavior was disregarded in the subsequent analysis of the data. The stiffness of each group was determined from the maximum slope of the stress‐strain curve in the linear elastic region.

*Statistical Analysis*: The testing data were described as the means and standard deviations for at least three samples. The mean value was statistically compared among the groups using a one‐sample *t*‐test. Probability values less than 0.05 were considered significant. A commercially available software program (Origin 8.5, Electronic Arts Inc., USA) was used for the statistical analysis.

## Conflict of Interest

The authors declare no conflict of interest.

## Supporting information

Supplementary

Supplementary

Supplementary
