# Supplementary material for: Osteoporotic Bone Recovery by a Highly Bone‐Inductive Calcium Phosphate Polymer‐Induced Liquid‐Precursor
Source: Adv Sci (Weinh). 2019 Aug 20;6(19):1900683. doi: 10.1002/advs.201900683 (PMC6774089; doi:10.1002/advs.201900683)
Supplement: Supplementary file 1 — Supplementary [file ADVS-6-1900683-s002.pdf]

Copyright WILEY-VCH Verlag GmbH & Co. KGaA, 69469 Weinheim, Germany, 2019.

Supporting Information

for *Adv. Sci.,* DOI: 10.1002/advs.201900683

Osteoporotic Bone Recovery by a Highly Bone-Inductive Calcium Phosphate Polymer-Induced Liquid-Precursor

*Shasha Yao, Xianfeng Lin, Yifei Xu, Yangwu Chen, Pengcheng Qiu, Changyu Shao, Biao Jin, Zhao Mu, Nico A. J. M. Sommerdijk, and Ruikang Tang**

Supporting Information

Osteoporotic bone recovery by a highly bone-inductive calcium phosphate polymer-induced liquid-precursor

*Shasha Yao, Xianfeng Lin, Yifei Xu, Yangwu Chen, Pengcheng Qiu, Changyu Shao, Biao Jin, Zhao Mu, Nico A. J. M. Sommerdijk, and Ruikang Tang**

Figures S1 to S11

Movies S1 to S2

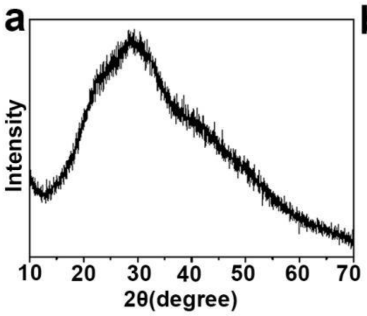

b

Elt.	Line	Intensity (c/s)	Atomic %
Al	Ka	46.70	0.621
Si	Ka	5,876.52	98.410
P	Ka	14.66	0.492
Ca	Ka	19.05	0.477
			100.000

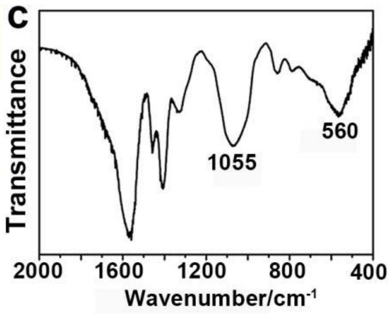

Figure S1. pXRD spectrum (a), EDS data (b), and FT-IR spectrum (c) of the obtained CaP-PILP.

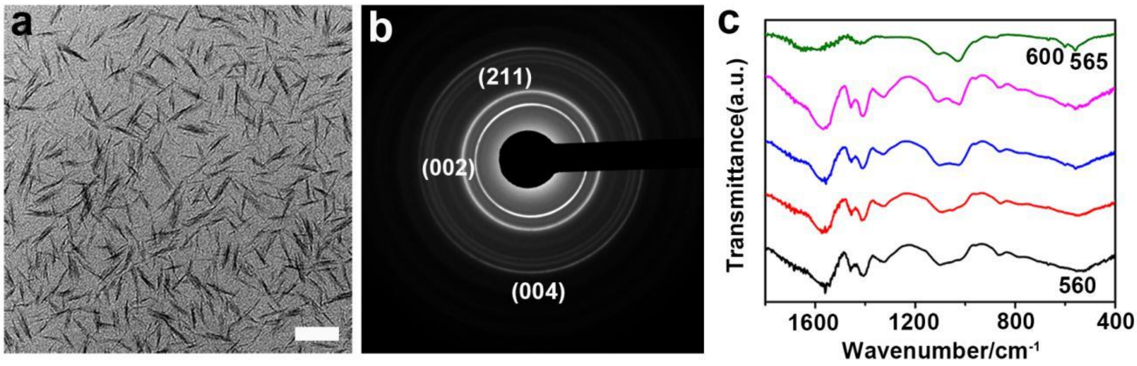

Figure S2. TEM image (a) and SAED image (b) of the solidified CaP-PILP. c) The FT-IR patterns show the evolution of CaP-PILP. 30 min (black line), 1 day (red line), 3 days (blue line), 5 days (pink line), and 7 days (green line). Scale bar: 20 nm.

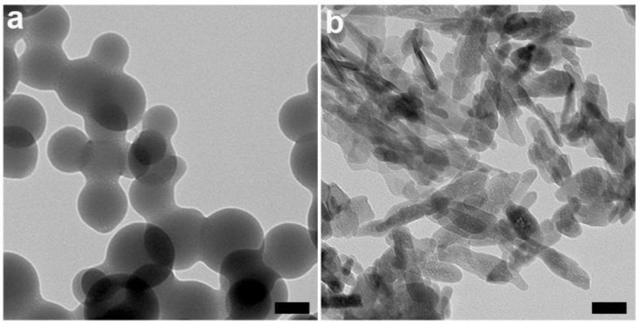

Figure S3. TEM images of the commercial ACP (a) and HAP (b) particles. Scale bars: 50 nm.

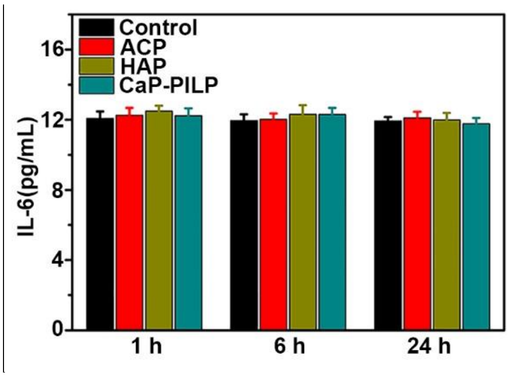

Figure S4. The expression of IL-6 in the control, ACP, HAP and CaP-PLP groups.

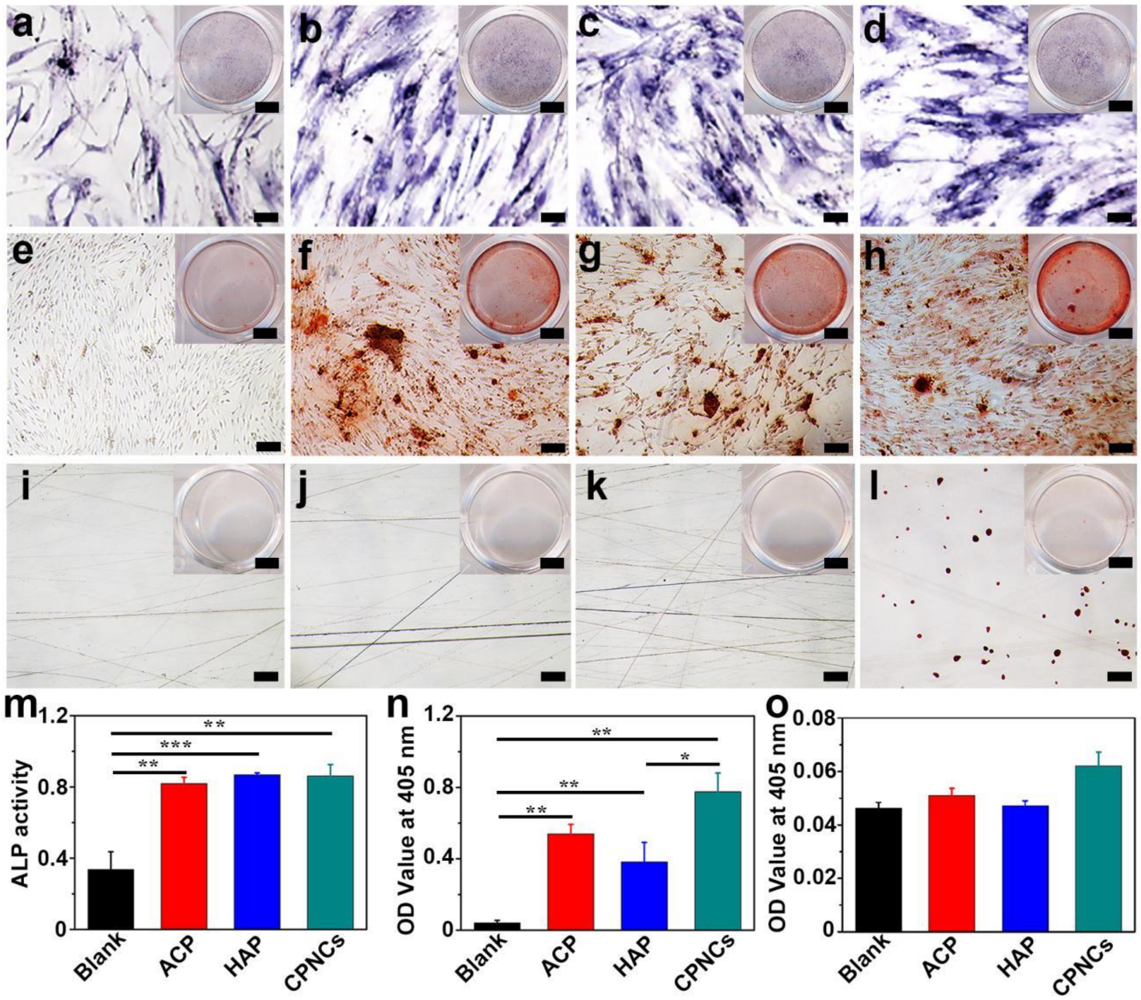

Figure S5. a – d) Expression of ALP activity of MSCs with blank (a), ACP group (b), HAP group (c), and CaP-PILP group (d) after incubation for 7 days. e – h) The ARS staining of the calcium deposition of MSCs with the blank (e), ACP group (f), HAP group (g), and CaP-PILP group (h) after incubation for 14 days. i – l) The ARS staining of the calcium deposition of the osteogenic medium with the blank (i), ACP group (j), HAP group (k), and CaP-PILP group (l) without MSCs after incubation for 14 days. m) ALP activity from MSCs for 7 days in osteogenic medium with the blank, ACP, HAP, and CaP-PILP groups. n) The OD value of the blank, ACP, HAP, and CaP-PILP groups with MSCs for 14 days. o) The OD value of the blank, ACP, HAP, and CaP-PILP groups. without MSCs for 14 days. Scale bars: 0.2 cm (insets in a – l), 200 μm (a – l). n = 3, $^{*}p < 0.05$.

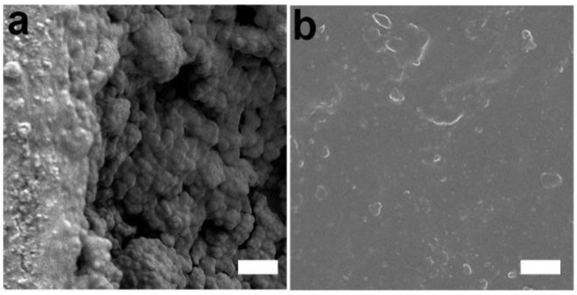

Figure S6. SEM images of HAP recovered bone (a) and healthy bone (b). Scale bars: 10 μm.

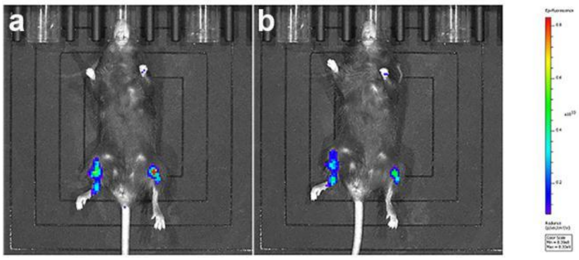

Figure S7. Permeability of calcein-stained CaP-PILP in osteoporotic tibia for 30 min (a) and 2 h (b).

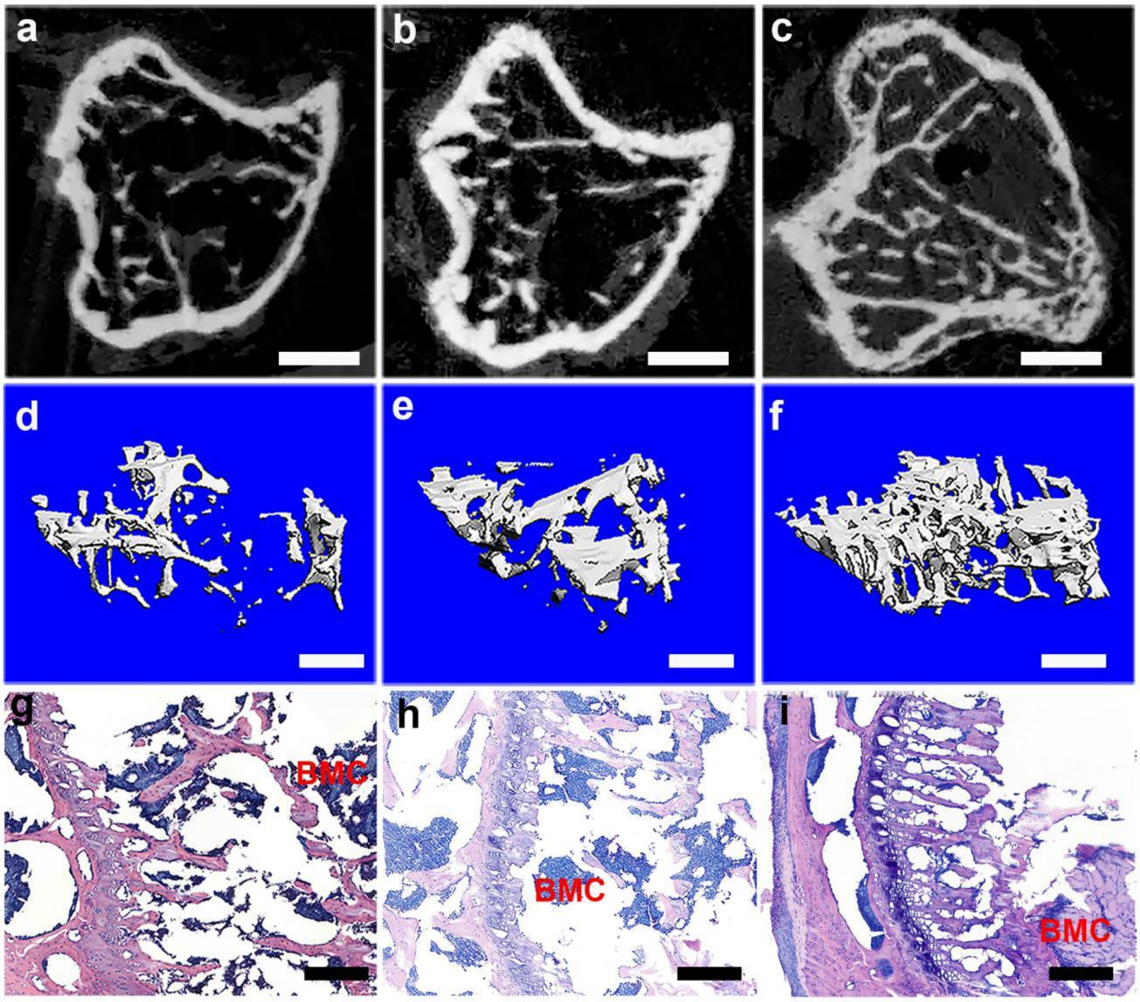

Figure S8. *In vivo* results of osteoporotic bone, PBS recovered bone, and healthy bone after 4 weeks. a – i) Representative 2D, 3D micro-CT and H&E staining of osteoporotic bone (a, d, and g), PBS recovered bone (b, e, and h), and healthy bone (c, f, and i) BMC: bone marrow cells. Scale bars: a – c, 100 μm; d – f, 300 μm; g – i, 200 μm.

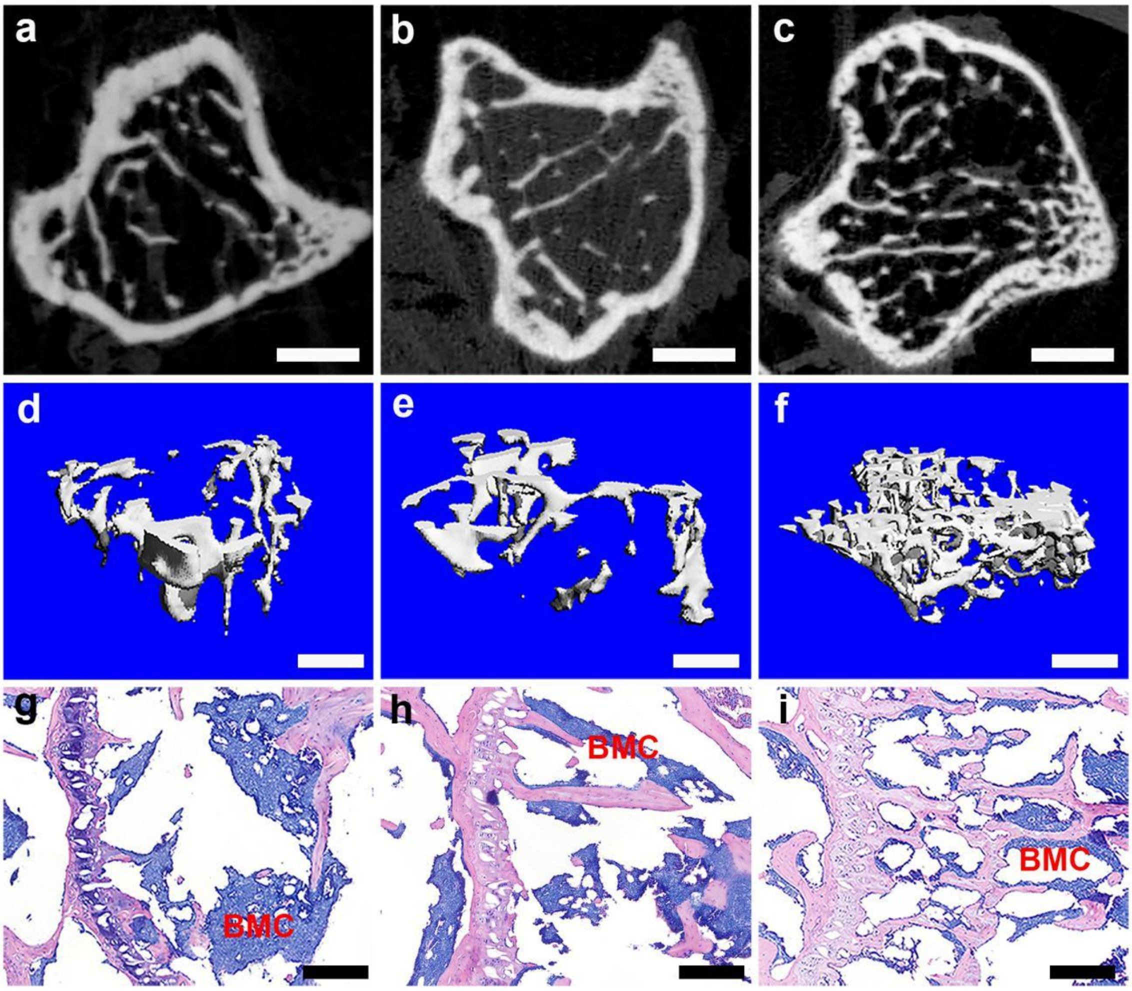

Figure S9. *In vivo* results of osteoporotic bone, PBS recovered bone, and healthy bone after 8 weeks. a – i) Representative 2D, 3D micro-CT and H&E staining of osteoporotic bone (a, d, and g), PBS recovered bone (b, e, and h), and healthy bone (c, f, and i) BMC: bone marrow cells. Scale bars: a – c, 100 µm; d – f, 300 µm; g – i, 200 µm.

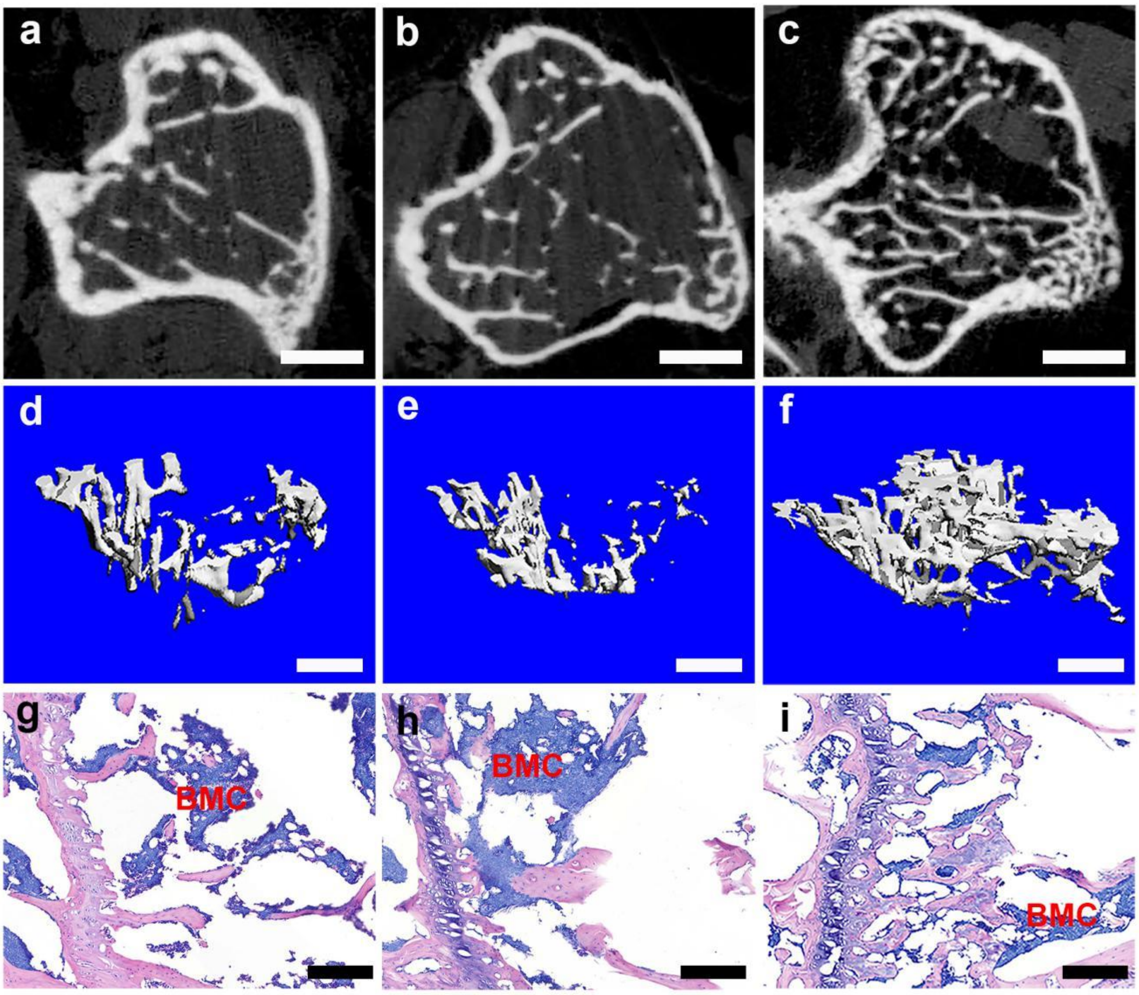

Figure S10. *In vivo* results of osteoporotic bone, PBS recovered bone, and healthy bone after 12 weeks. a – i) Representative 2D, 3D micro-CT and H&E staining of osteoporotic bone (a, d, and g), PBS recovered bone (b, e, and h), and healthy bone (c, f, and i) BMC: bone marrow cells. Scale bars: a – c, 100 μm; d – f, 300 μm; g – i, 200 μm.

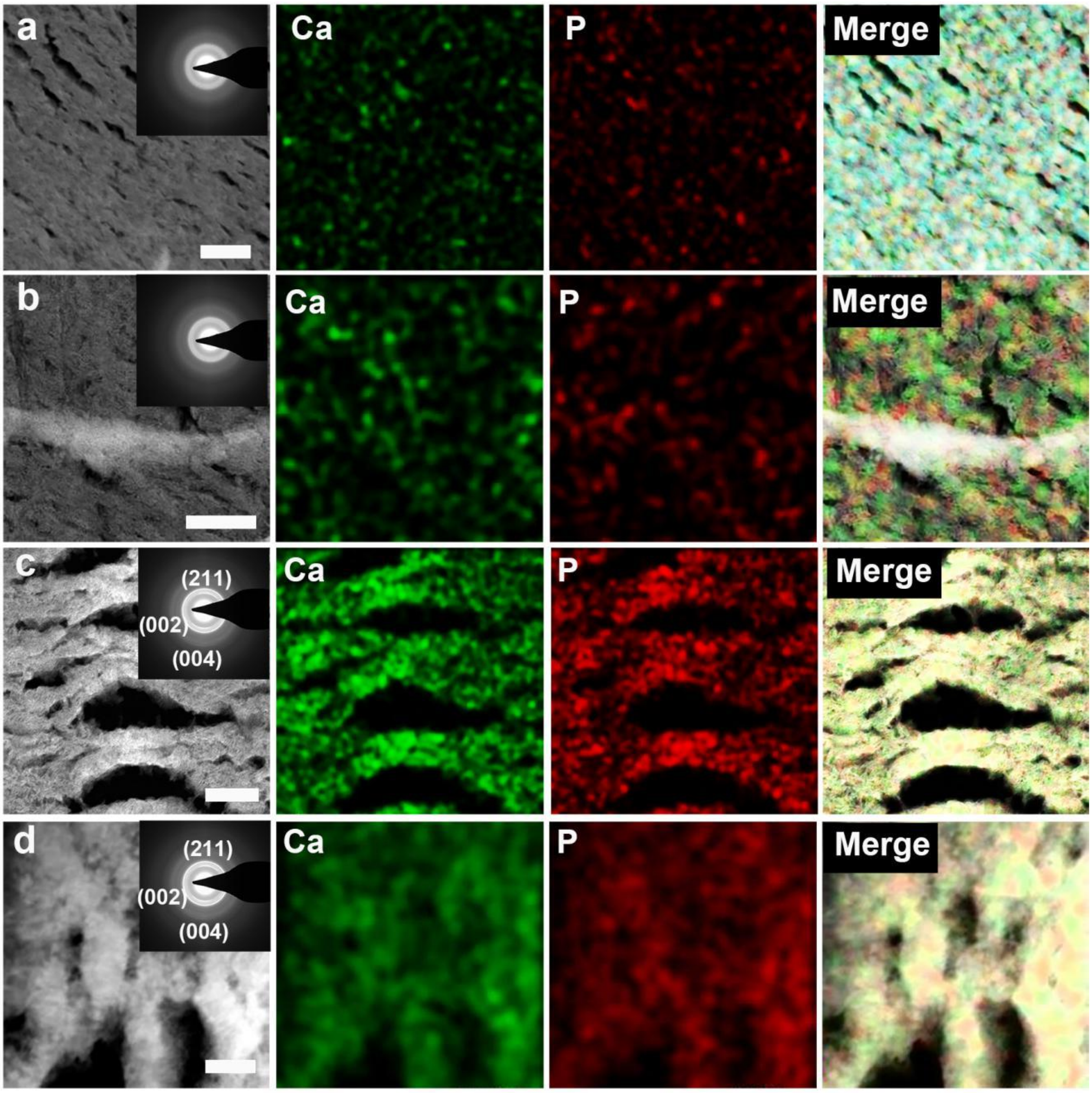

Figure S11. Element mapping of osteoporotic bone and recovered bones after 8 weeks. a – d) Element mapping of osteoporotic bone (a), PBS recovered bone (b), CaP-PILP recovered bone (c), healthy bone (d). SAED patterns of osteoporotic bone (inset in a), PBS recovered bone (inset in b), CaP-PILP recovered bone (inset in c), healthy bone (inset in d). Scale bars: a, 600 nm; b, 300 nm; c, 300 nm; d, 150 nm.
